# Deep *amoA* amplicon sequencing reveals community partitioning within ammonia-oxidizing bacteria in the environmentally dynamic estuary of the River Elbe

**DOI:** 10.1038/s41598-020-74163-0

**Published:** 2020-10-13

**Authors:** M. Malinowski, M. Alawi, I. Krohn, S. Ruff, D. Indenbirken, M. Alawi, M. Karrasch, R. Lüschow, W. R. Streit, G. Timmermann, A. Pommerening-Röser

**Affiliations:** 1 Department of Microbiology and Biotechnology, University of Hamburg, Ohnhorststr. 18, 22609 Hamburg, Germany; 2 Section Geomicrobiology, GFZ German Research Centre for Geosciences, Telegrafenberg, 14473 Potsdam, Germany; 3 Heinrich-Pette-Institute, Leibniz Institute for Experimental Virology, AG96 Technology-Platform, Martinistr. 52, 20246 Hamburg, Germany; 4 Hamburg Port Authority AöR, Neuer Wandrahm 4, 20457 Hamburg, Germany; 5 Bioinformatics Core, University Medical Center Hamburg-Eppendorf, Martinistr. 52, 20246 Hamburg, Germany

**Keywords:** Molecular ecology, Water microbiology, Freshwater ecology, Environmental monitoring, Hydrology

## Abstract

The community composition of betaproteobacterial ammonia-oxidizing bacteria (ß-AOB) in the River Elbe Estuary was investigated by high throughput sequencing of ammonia monooxygenase subunit A gene (*amoA*) amplicons. In the course of the seasons surface sediment samples from seven sites along the longitudinal profile of the upper Estuary of the Elbe were investigated. We observed striking shifts of the ß-AOB community composition according to space and time. Members of the *Nitrosomonas oligotropha*-lineage and the genus *Nitrosospira* were found to be the dominant ß-AOB within the river transect, investigated. However, continuous shifts of balance between members of both lineages along the longitudinal profile were determined. A noticeable feature was a substantial increase of proportion of *Nitrosospira*-like sequences in autumn and of sequences affiliated with the *Nitrosomonas marina*-lineage at downstream sites in spring and summer. Slightly raised relative abundances of sequences affiliated with the *Nitrosomonas europaea/Nitrosomonas mobilis*-lineage and the *Nitrosomonas communis*-lineage were found at sampling sites located in the port of Hamburg. Comparisons between environmental parameters and AOB-lineage (ecotype) composition revealed promising clues that processes happening in the fluvial to marine transition zone of the Elbe estuary are reflected by shifts in the relative proportion of ammonia monooxygenase sequence abundance, and hence, we propose ß-AOB as appropriate indicators for environmental dynamics and the ecological condition of the Elbe Estuary.

## Introduction

**Figure 1:**
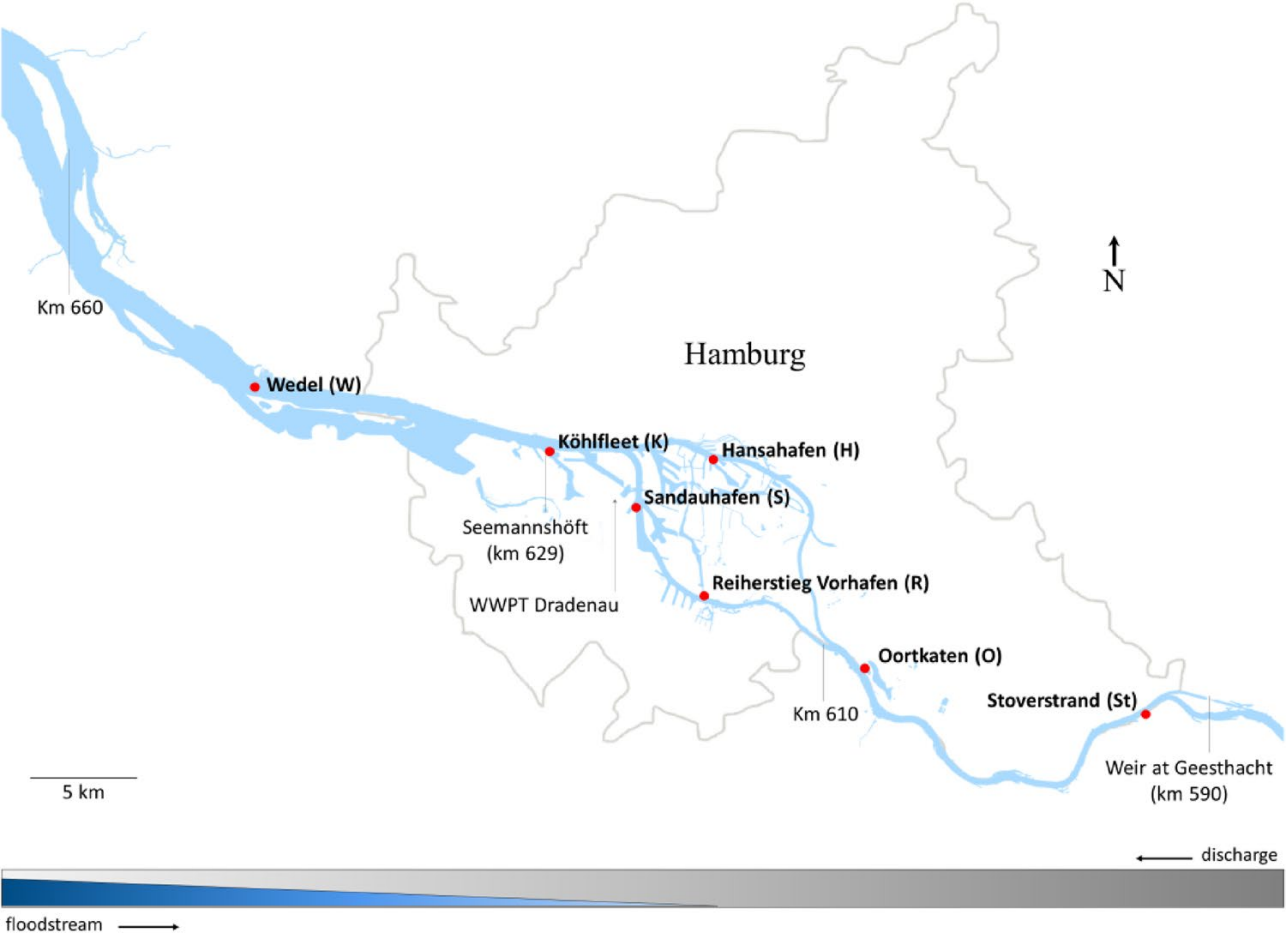
Map of the freshwater region of the Elbe Estuary showing the seven sampling sites (red dots) within a 46 km section along the Lower Elbe. The flow pattern at the bottom edge of the figure symbolizes the influence of discharge (grey) and influence of floodstream (blue) on particulate matter transport.

The Elbe Estuary is a highly dynamic environment. Its particular complexity results from both natural forces and anthropogenic influences. Of major relevance are the daily tides, as well as their interaction with headwater discharge causing continuous changes of influxes and effluxes of materials with effects on erosion, transport and sedimentation (Fig. 1). High water discharge which usually occurs in spring after snowmelt or due to flood events lead to low sedimentation of suspended matter, predominately of upstream origin. Low water discharge, however, result in increased sedimentation of material originating from marine or estuarine areas. Furthermore, the activities surrounding the Hamburg port, one of the largest ports in Europe, and the metropolitan region of Hamburg (e.g. various industries, ship traffic, discharge of waste water, dredging and disposal of sediments) affect the dynamics of biological, physical and chemical processes in the Lower Elbe to a considerable extent (HPA, annual reviews, https://www.hamburg-port-authority.de).

To display the multidimensional nature of the Elbe Estuary, a bioindicator-based approach was chosen, using lithotrophic ammonia-oxidizing bacteria (AOB) as model-group. Apart from the fact that other organisms or organism groups basically could function as models too, we selected AOB due to some prominent characteristics and advantages that, in our point view, predestined them for the above-mentioned issues. First, AOB fill a key-position in global nitrogen cycling and are therefore of particular ecological importance. Second, AOB are characterized by a common metabolism, a fact which enables us to study them solely on the basis of functional gene sequences. Third, AOB are phylogenetically well defined. Therefore, sequences of evolutionary markers can be assigned to different AOB-ecotypes. For this reason, among others, AOB have been proposed as “*E. coli* of Molecular Microbial Ecology” some time ago^1^, an idea, which we wish to renew by applying latest techniques.

The lithotrophic ammonia oxidation is the first and rate-limiting step in autotrophic nitrification, which encompasses the oxidative part of the global nitrogen cycle, the conversion of ammonia, via nitrite, to nitrate. Its environmental relevance is therefore obvious and well documented by numerous publications (for review, see, e. g.^1–4^). In relation to its ecological impact, only a manageable number of microorganisms is involved, which gives ammonia oxidation the characteristic of being a bottle neck process in the global nitrogen cycle. For more than a century AOB were believed to be the only lithotrophic ammonia-oxidizers in natural aerobic environments. However, since the discovery of ammonia-oxidizing Thaumarchaeota (AOA)^5^ and the recent finding that members of *Nitrospira,* a bacterial genus previously thought of as being specialized in nitrite oxidation, are able to oxidize ammonia to nitrate (Comammox)^6,7^ the perception of the lithotrophic ammonia-oxidation has changed. Subsequently, questions and controversial debates regarding niche differentiation, co-existence and competition between the above-mentioned groups of ammonia-oxidizing microorganisms (AOM) were in the focus of recent environmental studies^8–14^.

In contrast, the present study is consciously limited to the bacterial ammonia oxidizers (AOB) which are considered to belong to the most thoroughly investigated nitrifiers^15^. Furthermore, they can be regarded as one of the few cases in microbial ecology where pure cultures of nearly all recognized lineages of descent are available^16^. With the exception of the halophilic *Nitrosococcus* species^17–20^ and the recently proposed *Candidatus* Nitrosoglobus terrae isolated from acidic soils^19^ all AOB belong to the family Nitrosomonadaceae^21^ within the (recently proposed to be obsolete^22^) class Betaproteobacteria. These organisms are hereinafter designated as ß-AOB. Within this group, sufficient variation has evolved to permit exploitation of ammonia as energy source in the full range of aerobic environments. On the basis of resulting phenotypic differences 15 species in total have been described. Moreover, over a hundred pure cultures were isolated from various environments and accordingly investigated by laboratory experiments. Phylogenetic trees obtained by both 16S rRNA gene and *amoA* gene analyses revealed a consistent topology showing a pronounced sub-structuring with at least six distinct lineages of descent with sufficient depth of characterization. Members of individual lineages, in turn, share common features, obviously associated with corresponding environmental conditions. This provides the opportunity for comparisons to reveal causal relations between abundance of certain AOB-lineages and the environmental situation at specific sites.

Due to their ecological importance, their uniform metabolism, their physiological uniqueness and their monophyletic nature ß-AOB have been considered as “a model for molecular microbial ecology”^1^ and might provide us with ideal indicators for the above-mentioned objective.

In autumn 2013 we initiated a campaign to survey the colonization of surface sediments by ß-AOB at seven different sites of the Elbe Estuary in the course of 1 year. The objective was (a) to examine if environmental processes are reflected by the community composition of ß-AOB and (b) to check whether, in turn, changes in community composition might provide insights into the dynamics of the river transect studied. In this context, it is noteworthy, that current approaches monitoring estuarine dynamics are primarily of abiotic nature. A lot of attempts had been made to get insight into corresponding processes in the Elbe Estuary in recent decades e.g. the evaluation of clay minerals (smectite/kaolinite), of REE (rare earth elements), mass balancing as well as combined isotope/heavy metal concentration fingerprinting^23–26^. All these approaches, however, are often associated with particular challenges and difficulties due to the enormous versatility of the ecosystem.

The best way currently available to determine interactions between fluvial and tidal effects is the use of differences in contaminant level among fluvial and marine sediments. Here, especially cadmium and zinc are suitable candidates, because they show the steepest gradient^23–26^. This approach, however, can only give rough indications regarding the fluvial to marine mixing ratio since the upstream pollutant input varies substantially over time. Furthermore, one has to assume that there are no sources of contamination in the tidal area. Especially, in case the amount of contaminants decreases in future (which is highly desirable) new approaches will be necessary. We therefore see a need for alternative methods that can complement the existing approaches to examine sediment origin on shorter time scale (seasonally) and over shorter distances (e.g. in the vicinity of the WWTP).

Therefore, in this study spatio-temporal distribution of ß-AOB populations were monitored by high throughput sequencing of *amoA* amplicons. We tested the hypothesis that shifts in ß-AOB community structure would indicate the environmental dynamics and the ecological characteristics of the Elbe Estuary more reliable.

## Materials and methods

### Sampling sites and sediment collection

The River Elbe is one of the major waterways in Central Europe. With a length of about 1100 km it runs from the Czech Republic through Germany and flows into the North Sea 110 km northwest of Hamburg. It´s estuary starts at the weir in Geesthacht at river km 590 (applies to the German navigation kilometer whereby the German-Czech border is defined as kilometer 0).

About 20 km downstream at km 610 the River Elbe splits into two branches, the Northern- and the Southern-Elbe. In both branches the navigation channel is deepened down to 15 m for the ocean-going vessels to get access to the port basins. Consequently, the water volume increases from 15.5 Mio m^3^ at km 608 to 47 Mio m^3^ in the Northern and 43 Mio m^3^ in the Southern Elbe. At river km 625.7 the Northern and the Southern Elbe rejoin to form a broad riverbed with a volume of 99 Mio m^3^. The volumes mentioned are related to average tidal conditions during flood tide and a headwater discharge of 350 m^3^/s (data source: Hamburg Port Authority, unpublished). Further downstream at river km 683 the freshwater region of the Elbe Estuary ends (Fig. 1).

Surface sediment samples (0–5 cm) were collected on 30th August and 2nd September, 2013 (autumn), 13th and 16th December, 2013 (winter), 28th and 29th April, 2014 (spring) and on 16th and 17th July, 2014 (summer) from seven distinct sites. At each of the sites five samples were taken with a Van Veen grab and combined to a mixed sample. All sampling-sites were located in the expanded freshwater region of the Elbe Estuary: “Wedel” (W, 9° 40′ 17.584″ E, 53° 34′ 2.076″ N, river km 643–644), “Köhlfleet” (K, 9° 52′ 33.773″ E, 53° 32′ 20.589″ N, river km 629), “Hansahafen” (H, 9° 59′ 25.641″ E, 53° 32′ 8.313″ N, river km 621,5), “Sandauhafen” (S, 9° 56′ 10.888″ E, 53° 31′ 2.601″ N, river km 621.5), “Reiherstieg-Vorhafen “ (R, 9° 59′ 1.838″ E, 53° 28′ 41.485″ N, river km 615.5), “Oortkaten” (O, 10° 5′ 51.432″ E, 53° 26′ 43.077″ N, river km 607), and “Stover Strand” (St, 10° 17′ 33.685″ E, 53° 25′ 32.640″ N, river km 597).

The sampling sites were selected in areas where fine textured sediments were recently deposited. The sites Stover Strand and Oortkaten are situated upstream the port of Hamburg at the inland waterway depth of 2.0 to 2.5 m. The sampling sites Reiherstieg-Vorhafen, Sandauhafen, Hansahafen and Köhlfleet are located in the front part of port basins at a required water depth of about 15 m. Due to the cross-sectional widening of the water column at these sites, sediment lenses form which have to be dredged regularly. The site Wedel is located in the navigation channel below the port of Hamburg in a sediment trap at about river km 642 to 644, which is also dredged regularly.

### DNA extraction

Aliquots of the sediment samples were cooled down to 4 °C, immediately after sampling and stored at − 80 °C after arrival in laboratory.

DNA was extracted from 250 mg sediment sample using the “POWER Soil DNA-Isolation Kit” from MO BIO Laboratories according to manufacturer's instructions with additional proteinase K-treatment (0.5 mg per sample). The yield and purity of the isolated DNA was estimated spectrophotometrically. Integrity was visualized by ethidium bromide staining in 0.8% agarose gel electrophoresis.

### Amplicon-sequencing of amoA genes

For amplicon-sequencing we used the primers 5ʹ-TCG TCG GCA GCG TCA GAT GTG TAT AAG AGA CAG CGG GHT TYT ACT GGT GGT-3ʹ (forward primer) and 5ʹ-GTC TCG TGG GCT CGG AGA TGT GTA TAA GAG ACA GCC CCT CKG SAA AGC CTT CTT-3ʹ (reverse primer) originally described by Rotthauwe et al.^27^. PCR reaction mixtures contained 100 ng of template DNA per μl, 0.2 mM of each of the four desoxynucleoside triphosphates, 1.5 mM MgCl_2_, 1 μM of each primer, and 2.5 U of Taq DNA polymerase. Thermocycling conditions included 30 s of denaturation at 95 °C, 30 s of primer annealing at 47 °C, and 30 s of primer extension at 72 °C. This cycle was repeated 30 times. After a PCR clean-up step, a second PCR attaches dual indices and Illumina sequencing adapters following the manufacturer´s instructions of the “Nextera XT Index Kit”. PCR products were cleaned up using the Gel/PCR DNA Fragments Extraction kit (Qiagen, Hilden, Germany) and sequenced on an Illumina MiSeq sequencing platform in 2 × 250 bp paired-end mode. Between 0.145 and 1.083 million read pairs were obtained for each sample (median: 0.483 M).

### Data processing and analysis

Primer sequences were removed from sequence reads using cutadapt (v1.9.1)^28^. Reads not containing primer sequences were discarded. Overlapping paired reads were then merged with VSEARCH (v.2.4.3)^29^. VSEARCH was also used for subsequent steps unless indicated otherwise. Low quality bases (Phred quality score < 10) were trimmed from the 3′-end of reads. Merged sequences were discarded if the overlapping region was shorter than 10 bp or if there was more than a single mismatch in the overlapping region. 93.06% of all remaining merged sequences were 492 bp long and 95.5% were at least 480 bp long. The sequences were converted from FASTQ to FASTA format. A subset of these sequences was used to create reference clusters. Sequences in this subset were required not to contain the letter 'N' and not to be expected to contain more than a single erroneously called base. The subset was than reduced to a set of unique sequences and each of these sequences was annotated with the number of individual sequences it represented. Sequences observed only once were discarded and the remaining sequences were clustered at 97% sequence similarity to account for technical variation. Finally, all sequences were aligned to the resulting clusters to estimate OTU abundance and to generate an OTU table. For this step, sequence similarity had to be at least 97% over a region of at least 250 bp. For the taxonomic assignments, the centroids of the OTUs were aligned to a reference database of *amoA*/*amoB* sequences obtained from the NCBI data base (May/2018). For this step, sequence similarity had to be at least 85%. For multivariate statistics CANOCO 5^30^ and PAST3^31^ software was used.

### Data deposition

Sequence data has been deposited at the European Nucleotide Archive (https://www.ebi.ac.uk/ena) under the accession number PRJEB28821.

### Real-time PCR

The abundance of bacterial *amoA* genes was quantified by real-time PCR (RT-PCR) using SYBR™ Select Master Mix for CFX (Applied Biosystems by Thermo Fisher Scientific) on a QuantStudio 3 detection system (Applied Biosystems by Thermo Fisher Scientific) with the use of the oligonucleotide pair amoA189/amoA337^32,33^. All RT-PCR assays were performed in 16 µL reaction mixtures with the following reaction composition: 8 µL SYBR™ Select Master Mix, 0.4 µL of each primer (500 nmol) and 2.4 µL DNA-template at a concentration of 1 ng/µL. The thermal profile for the real-time PCR was as follows: An initial denaturation step for 3 min at 93 °C followed by 40 cycles of 15 s at 95 °C, 20 s at 58 °C and 20 s at 72 °C. To show the proportion between bacterial *amoA* copy number and C_T_ value a standard curve with a tenfold serial dilution of known *amoA* copy numbers was generated. The standard curve covered a range from 10^3^ to 10^8^ copies of bacterial *amoA* genes. The relationship between C_T_ value and gene copy numbers was linear over 6 orders of magnitude (R^2 ^= 0.97; Eff% = 98%; y = − 3.36 × log_10_ x + 44.23). All reactions, including standards, positive and negative controls, were run in triplicates and a melting curve analysis was plotted after each run to check the product specificity.

### Determination of sediment characteristics

The determination of sediment parameters was carried out by commercial laboratories. The sediments were freeze-dried and passed through a 2 mm sieve. Total organic carbon (TOC) was analyzed according to DIN EN 13137. To remove calcium carbonate, hydrochloric acid was added to the milled samples. The samples were then combusted in an oxygen-rich atmosphere and the carbon dioxide was measured using an infrared detector. In order to determine the content of heavy metals samples were subjected to aqua regia digestion and analyzed for arsenic, lead, cadmium, chromium, copper, iron, nickel and zinc with ICP-MS (DIN CEN 16171). Mercury was analyzed in the aqua regia digests with AAS according to DIN EN 1483. Grain size distribution was determined by wet sieving with additional ultrasonic dispersion. The fraction < 20 µm was collected and also subjected to analysis of heavy metals.

For collection of eluates the water content of field moist sediment samples was supplemented by the addition of synthetic freshwater to a constant sediment to water ratio of 1:3 (w/w). Because of the high water content only small amounts of water had to be added so that the eluent consisted mostly of pore water. The samples were shaken for 24 h at room temperature in an overhead shaker at 7 rpm. Eluates were collected by centrifugation (17,000*g*, 20 min). Directly after centrifugation the eluates were analyzed for oxygen, salinity and pH-value and chemical parameters (ammonium, nitrate, nitrite, nitrogen and DOC). The determination of chemical parameters in the eluates was carried out with standard methods. Nitrate and nitrite were determined according to DIN EN ISO 10304-1 by liquid chromatography. Total nitrogen was measured photometrically according to DIN EN ISO 11905-1 after oxidative decomposition and reaction with 2,6-dimethylphenol in a sulfuric acid/phosphoric acid mixture. Ammonium reacts with hypochlorite and salicylate in the presence of sodium nitroprusside as catalyst to form a blue indophenol that was measured photometrically according to DIN38406-E5. DOC was measured according to DIN EN 1484 after filtration with a RC filter (0.45 µm). For determination of the organically bounded carbon, a thermic-catalytic oxidation is generated in the TC-reactor. The determination of the inorganically bounded carbon took place in the TIC-reactor via phosphoric acid. The free carbon dioxide was thereafter detected in an infrared measuring cell.

## Results

### Diversity and community composition

**Figure 2:**
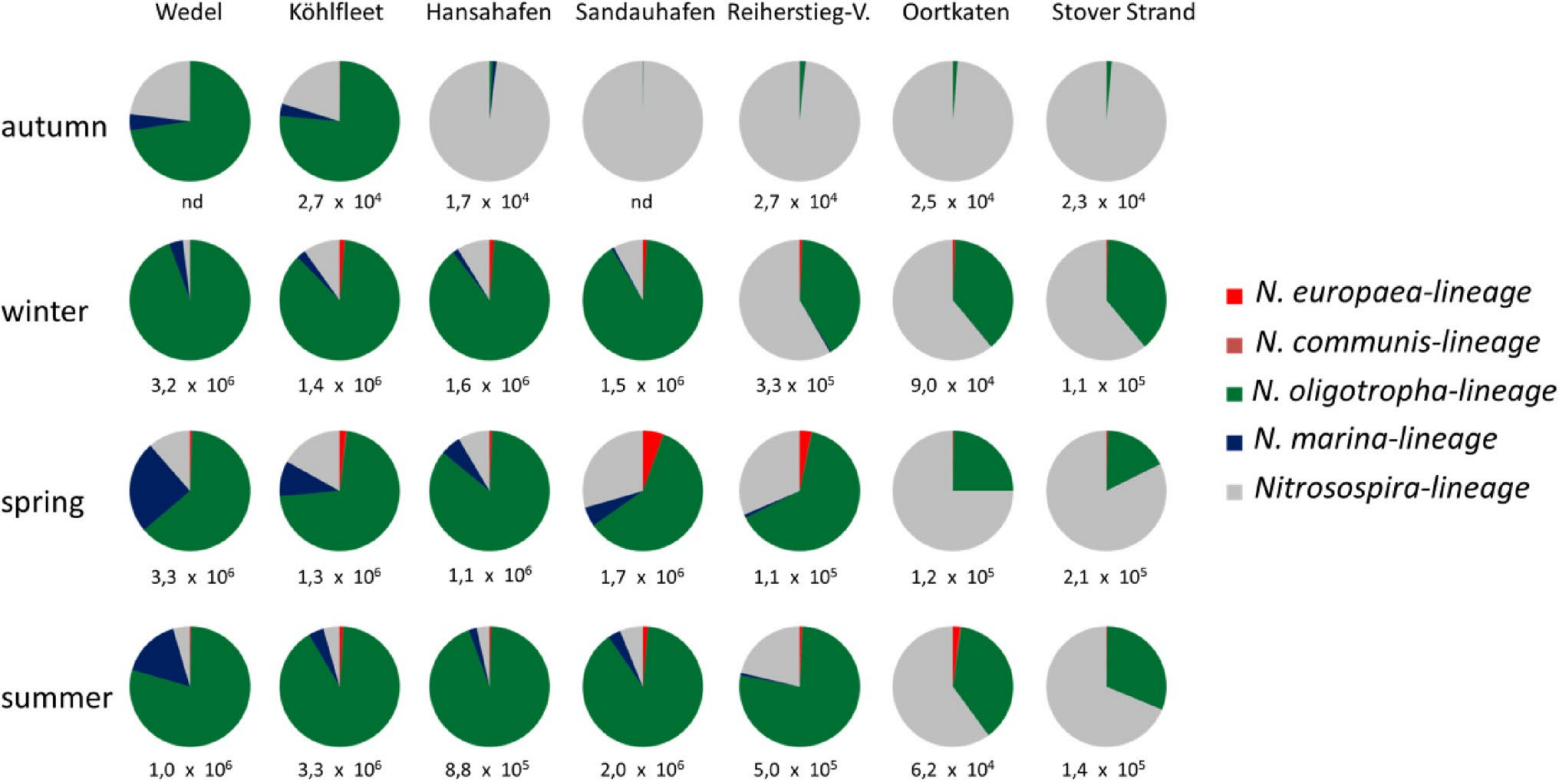
Relative abundances of the different ß-AOB lineages over time and space based on the proportional frequencies of *amoA*-sequences. Different colors indicate the distinct lineages: *Nitrosomonas oligotropha*‐lineage (green), *Nitrosospira*-lineage (grey), *Nitrosomonas marina*-lineage (blue), *Nitrosomonas europaea/ Nitrosomonas mobilis*-lineage (red), *Nitrosomonas communis*-lineage (invisible due to low abundance). The numbers below the pie charts indicate total amount of *amoA* copies per gram of sediment (nd: not determined).

The population structure of ß-AOB was analyzed by deep sequencing of *amoA* amplicons. A total of 13.8 M paired-end reads were sequenced, of which 13.3 M sequences remained after trimming and merging the forward and reverse reads. An average of 476,000 reads was obtained for each sample. In total 4.8 M reads were assigned to 75 OTUs (Table S1). The OTUs were sorted to five lineages, namely: The *Nitrosospira*-lineage, the *Nitrosomonas oligotropha*-lineage, the *Nitrosomonas marina*-lineage, the *Nitrosomonas europaea/Nitrosomonas mobilis*-lineage and the *Nitrosomonas communis*-lineage (Fig. 2).

The majority of sequences were affiliated to the *Nitrosomonas oligotropha*-lineage and the *Nitrosospira*-lineage. Sequences affiliated with the other three lineages were found to a far lesser degree.

It is notable that the relative abundance of *Nitrosospira*-like sequences decreases from the upstream to the downstream sites of the river Elbe. Members of this lineage appear to form the numerically dominant population of the ß-AOB at the River Elbe sites Hansahafen (98.1%) , Sandauhafen (99.8%), Reiherstieg-Vorhafen (98.4%), Oortkaten (98.7%), and Stover Strand (98.5%) in autumn, at Reiherstieg-Vorhafen (58.4%), Oortkaten (61.0%), and Stover Strand (61.0%) in winter and at Oortkaten (75.0%, 60.1%) and Stover Strand (82.3%, 68.8%) in spring and summer, respectively.

In an opposite manner this applies accordingly to the sequences affiliated with the *Nitrosomonas oligotropha*-lineage which were most frequently observed at the downstream sites Wedel (63.3–94.3%) and Köhlfleet (71.7–90.5%) at all seasons as well as at Hansahafen (85.4–94.0%) and Sandauhafen (59.6–88.9%) apart from autumn.

A similar pattern, although less pronounced, was seen among the sequences related to the *Nitrosomonas marina*-lineage, most abundant in spring and summer at site Wedel (24.8% and 16.0%).

Signatures of members of the *Nitrosomonas europaea*-lineage were found at Sandauhafen and its neighbouring sites particularly in spring (3.1–5.6%).

The frequency of sequences related to the *Nitrosomonas communis*-lineage were in the per thousandths range. Furthermore, members of this lineage were found to occur only sporadically at individual sites (Table S1).

### Correlation between the community structure and environmental parameters

The sediments can be described as mostly fine textured with a < 63 µm grain size fraction ranging from 38 to 97% (Table S2). They cover a relatively wide range of organic matter content (2.0–7.3% TOC). The heavy metal concentrations vary in a range that is typical for this part of the tidal Elbe^25,26^. The sampling sites upstream the port, have on average higher heavy metal contents than the downstream ones. This holds true especially for cadmium and zinc and, to a lesser extent, for mercury and copper (Table S2).

The dissolved inorganic nitrogen (DIN) in the eluates consists almost exclusively of ammonium and it is closely correlated to the total nitrogen content (TN) of the sediments (r^2^ = 0.76***, n = 28). The highest ammonium concentration on all sampling dates is found at the site Reiherstieg-Vorhafen (R) with values of 6 to 11 mmol NH_4_-N/l (Table S2). Though these values are relatively high, they are in line with DIN concentrations measured in pore waters at this site in previous years (4 to 16 mmol NH_4_-N/l) and can therefore be considered typical.

**Figure 3:**
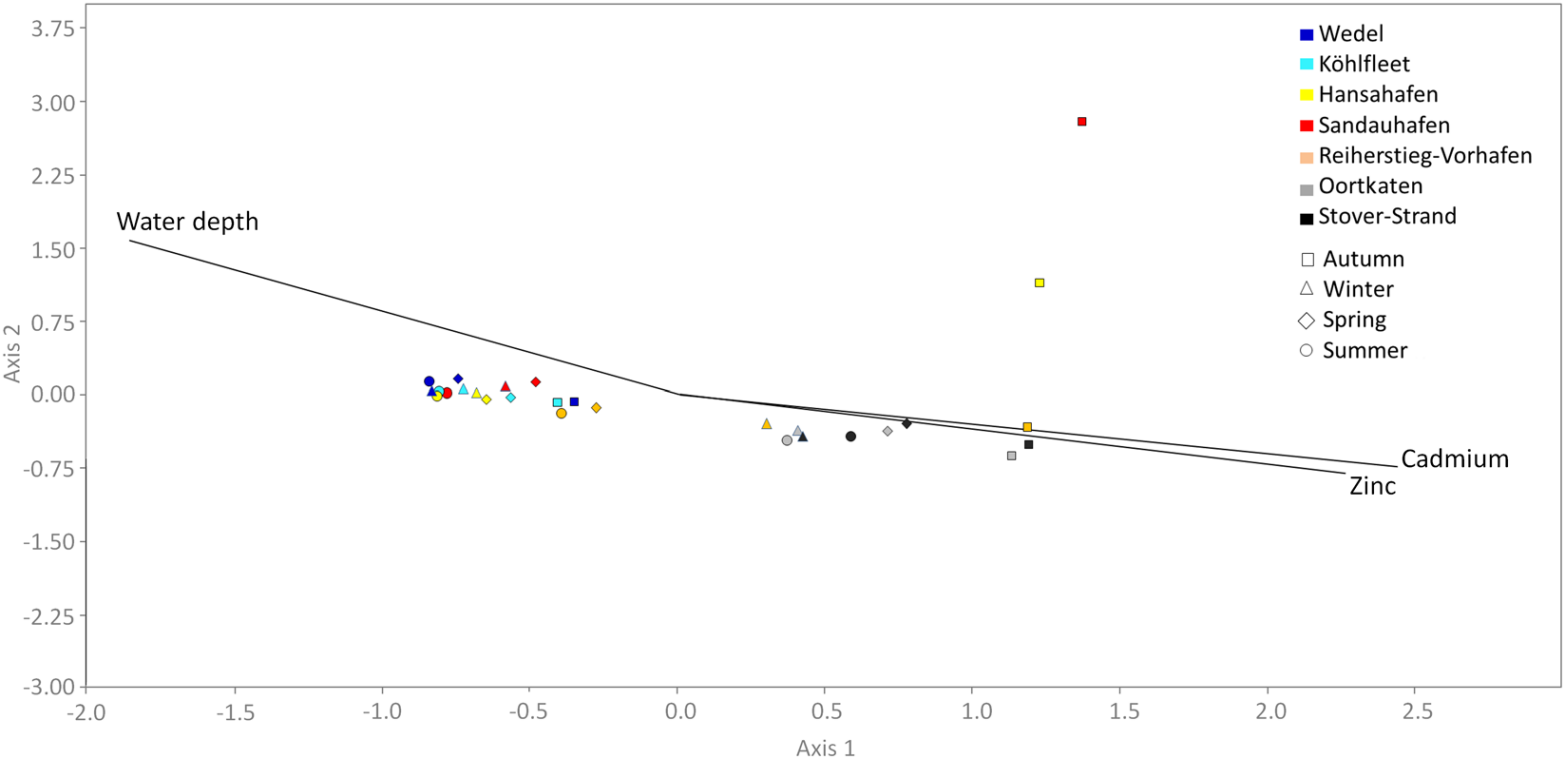
Canonical correlation analysis of ß-AOB community composition and environmental parameters showing that the communities form two clusters with exception of the autumn samples from Sandauhaufen (S) and Hansahafen (H). If the Bonferroni corrected p_adj_ was < 0.05, a given parameter was included. Cadmium, zinc and water depth explain 42.7% (adjusted) of the compositional variation among the analyzed sites which is interpreted as a co-correlation caused by the coupled transport of ß-AOB and heavy metals along the river transect, investigated. Analyses are based on *amoA* amplicons.

**Figure 4:**
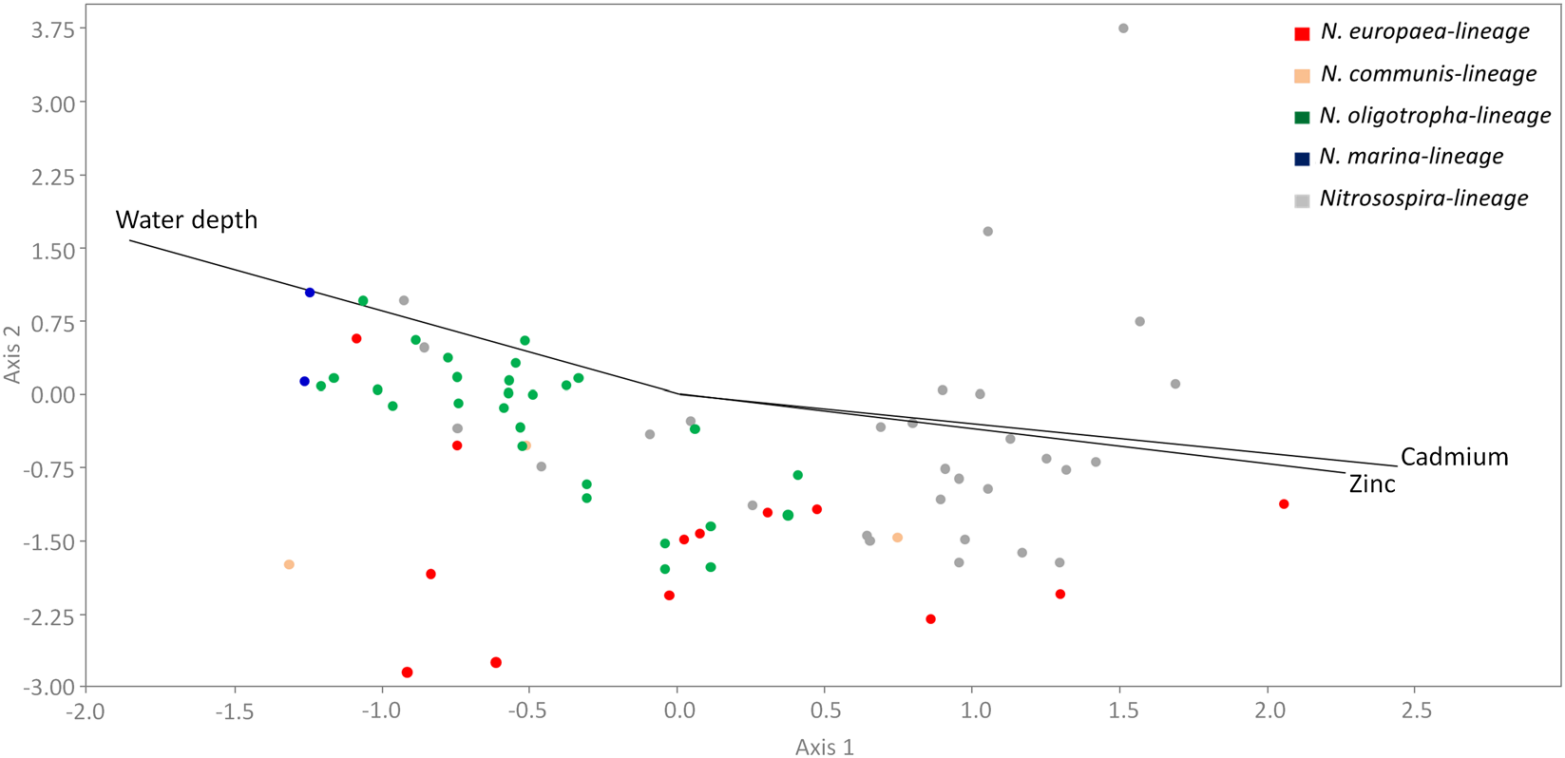
Canonical correlation analysis of the of ß-AOB community composition on OTU level and environmental parameters. If the Bonferroni corrected p_adj_ was < 0.05, a given parameter was included. The two clusters of communities found at the different sites (Fig. 3) are mainly explained by the relative abundance of OTUs related to the *Nitrosospira*-lineage and the *Nitrosomonas* oligotropha-lineage. Cadmium, zinc and water depth explain 42.7% (adjusted) of the compositional variation among the analyzed sites which is interpreted as a co-correlation caused by the coupled transport of ß-AOB and heavy metals along the river transect, investigated. Analyses are based on *amoA* amplicons.

The relationship between the ammonia-oxidizing community and environmental parameters was examined by canonical-correlation analyses (CCA) (Figs. 3, 4, S1 and S2). Independently from the season, the communities from Stover Strand (St) and Oortkaten (O) form one cluster (Fig. 3). The communities observed at the other sites are forming a second cluster, with exception of the autumn samples from Sandauhaufen (S) and Hansahafen (H) (Fig. 3). The two clusters are mainly explained by the relative abundance of OTUs related to the *Nitrosospira*-lineage and the *Nitrosomonas oligotropha*-lineage (Fig. 4). A forward selection of all analyzed environmental parameters (Table S2) revealed that the content of cadmium and zinc as well as the water depth (at sampling site), showed significant correlations (Bonferroni corrected p_adj_ < 0.05) to the community structures (Figs. 3 and 4). Cadmium (27.1%), which was recently proposed as indicator for marine to fluvial mixing ratio^25^, zinc (10.3%) and water depth (11.7%) explain together 42.7% (adjusted) of the compositional variation among sites, investigated.

### Quantification of amoA gene copies

The total numbers of *amoA* copies varied between 1.7 × 10^4^ and 3.3 × 10^6^ per gram of sediment depending on site and season (Fig. 2). It also noted that the copy numbers of the four downstream located sites (Wedel, Köhlfleet, Hansahafen, Sandauhafen) appeared to be approximately by one order of magnitude higher than those of the upstream sites (Reiherstieg-Vorhafen, Oortkaten, Stoverstrand). Furthermore striking is the low and relative constant number of *amoA* copies in autumn at all sites (1.7 × 10^4^ to 2.7 × 10^4^).

## Discussion

The aim of the present study was to prove the assumption that the community composition of ß-AOB might indicate the environmental dynamics of river habitats, exemplarily examined at the freshwater region of the Elbe Estuary. Analysis on the basis of *amoA* amplicon sequences revealed pronounced temporal and spatial differences of the community structure along the river transect, investigated which were interpreted as a result of estuarine dynamics, influenced by seasonal variations and specific meteorological events. Potential causal links between distribution and ecophysiological properties of the respective ammonia-oxidizers are discussed below.

*AmoA*-sequences assignable to the *Nitrosomonas oligotropha*-lineage and the *Nitrosospira*-lineage were most commonly observed in our study, followed at a distance by those of the *Nitrosomonas marina*- and the *Nitrosomonas europaea*-lineage. Signatures belonging to the *Nitrosomonas communis*-lineage were found most infrequently.

The *Nitrosomonas oligotropha*-lineage is represented by the species *N. oligotropha* and *N. ureae* as well as several genetically defined but not yet described species^34^. Most strains of this lineage were obtained from rivers and lakes, only few originate from oligotrophic, often moderately acidic soils^2^. Accordingly, all turned out to be sensitive against increasing salt concentrations. High substrate affinity and the ability to utilize urea as ammonia source are common features of isolates investigated until now^35,36^. As demonstrated by molecular approaches members of the *N. oligotropha-*lineage, are widely distributed in diverse aquatic environments^2,37^. These also include engineered habitats, such as wastewater treatment systems, which however are characterized by low-ammonia influents^38–41^, biofilm reactors^42^ or drinking water distribution systems^43^. A further characteristic feature is their capability of self-flocculation, which is usually interpreted as adaptation to the conditions of river habitats^44^. Recent investigations have indicated limnic and estuarine environments to be the natural habitats for members of the *N. oligotropha*-lineage, which is in accordance with our finding. Using denaturing gradient gel electrophoresis (DGGE) of PCR-amplified 16S rRNA gene fragments and subsequent sequencing (batch cultures, DGGE, sequencing) Bollmann and Laanbroek found *N. oligotropha* and *N. ureae* like bacteria to be the most ubiquitous AOB in the freshwater area of the River Schelde Estuary^45^. Sahan and Muyzer investigated three different intertidal sites of the same estuary by a similar approach^15^. These authors mentioned high abundances of sequences affiliated with the *N. oligotropha*-lineage in the brackish water region of the River Schelde. Investigating the impact of Paris wastewater effluents on the nitrifying communities in the lower River Seine, Cebron and Coworkers also reported that the majority of AOB detected belong to the *Nitrosomonas oligotropha*-lineage both upstream and downstream of the effluent output^46^.

A recent study on ß-AOB community responses to environmental gradients in surface sediments in the Pearl River Estuary also reports a dominant role of *N. oligotropha-*like organisms^47^. However, these authors interpreted their observations in connection with the eutrophication status of the waters investigated and proposed members of the *N. oligotropha*-lineage as potential bioindicators for pollution or wastewater input into aquatic environments. Against the background of the ecophysiological properties of the cultured representatives, members of the *N. oligotropha*-lineage are generally considered as typical AOB in aquatic environments low in ammonia^48^. Thus, it is more likely that *N. oligotropha-*sequences are present despite—and not because of the degree of pollution of the respective waters.

The *Nitrosospira*-lineage comprises the currently accepted genus *Nitrosospira* and the two previously described genera *Nitrosovibrio*, and *Nitrosolobus*. Although clearly distinguishable from each other by morphology, these genera represent a phylogenetically young group^2,37^. Four species are described within this lineage, *Nitrosospira briensis*^49,50^, *Nitrosospira lacus*^51^, *Nitrosovibrio (Nitrosospira)tenuis*^52^ and *Nitrosolobus (Nitrosospira) multiformis*^53^ and several further defined but unnamed species excist. All members of this lineage form at least six clusters whose phylogenetic node support is regarded to be low. Accordingly, cultures of this lineage share many common features and show with respect to their ecophysiological properties several similarities to those of the *Nitrosomonas oligotropha*-lineage. Ureolytic activity is a very widespread capacity but does not occur consistently. Representatives of this lineage are commonly regarded as the most ubiquitous AOB in nature^54–56^. They were frequently detected in untreated oligotrophic soils, like forest soils^57^ and grassland soils^58^, as well as in freshwater habitats^59–61^. Strains have been isolated from rocks, stone buildings^62^, and even, uniquely among the ammonia oxidizers from acidic soils^63^. Beyond that, sequences affiliated with the *Nitrosospira*-lineage were continually detected in marine or seawater influenced habitats^64–68^ . These sequences form distinct subgroups within this lineage, for which no cultured representatives exist, the 16S rRNA-based *Nitrosospira*-cluster 1^64^ as well as a yet non-specified *amoA*-sequence-based cluster. Marine or estuarine *Nitrosospira*-associated sequences were, amongst others, frequently found close to the mouth of estuaries, the Ythan Estuary, on the east coast of Scotland^69^, the Schelde Estuary^15^ and the Elkhorn Slough Estuary, California^70^ just to name a few examples. In contrast, *Nitrosospira*-like sequences detected by Cébron et al.^46^ preferential originated from upstream sites of the Lower Seine. This observation confirms our findings within the Elbe Estuary where the majority of sequences at the upstream sites belong to the *Nitrosospira*-lineage. Remarkably, these sequences were unexceptionally affiliated with cultured, phenotypically characterized nitrosospiras. According to our estimate the tremendous increase of the relative abundance of *Nitrosospira*-associated sequences in autumn 2013 can be plausibly explained, since abnormal rainfall followed by considerable flooding of the middle reach of the Elbe has taken place over the previous summer (Figures S3 and S4). As a result, large quantities of suspended solids identified as terrestrial organic material^71^ were transported downstream into the estuary and beyond into the adjacent German Bight^72^. These observations support our hypothesis, that an increasing proportion of the *Nitrosospira*-lineage-abundance displays fluvial influences. From that perspective, members of the *Nitrosospira*-lineage, with the exception of the marine, non-cultured ones, might serve as appropriate indicators for emissions of suspended matter originated from the inland reaches and possibly represent terrestrial organisms introduced into the waterbody by continuous soil leaching and as a result of heavy rainfall and floods^37,46^. This assumption is also supported by correlation analyses between community structure and environmental parameters in our present study showing strong impact of factors which reflect fluvial to marine transitions on community composition of ß-AOB (Figs. 3, 4, S1 and S2).

Members of the *Nitrosomonas marina*-lineage, these include *N. marina* and *N. aestuarii* as well as a further, yet unnamed species represented by the strain *Nitrosomonas* sp. Nm 51^73^, are typical sea water inhabitants^2^. All isolates of these species originate from marine environments^2,35^. Accordingly, cultures so far investigated turned out to be obligate halophiles showing optimal growth in a range of 300–400 mM NaCl. With regard to the low ammonia concentrations in their natural environments members of this lineage reveal remarkably high Ks values of their ammonia-oxidizing system and, furthermore, the ability to use urea as ammonia source^2,35^. The occurrence of *Nitrosomonas marina*-lineage associated sequences may generally be interpreted as a result of marine influences on the respective sites. In this context Nacke and coworkers detected respective sequences during their studies on the influence of seawater flooding on soil ammonia oxidizer communities from three German Halligen Nacke et al.^74^. Laanbroek and coworkers proved a correlation between estuarine water flooding of impounded mangrove forests and the abundance of *N. marina*-like AOB in the Indian River Lagoon, a coastal estuary on the east coast of Florida^75^. In the Schelde Estuary *Nitrosomonas marina*-lineage associated sequences were reported to be present at several sites along the salinity gradient, but most abundant at brackish sites^15,45^. Although, all sites investigated in our study can be considered as limnic we found signatures of the *Nitrosomonas marina*-lineage, having greatest frequencies of occurrence downstream at the sampling sites Wedel and Köhlfleet and declining relative abundances in direction of the upstream located sites. In winter corresponding signatures were found up to Sandauhafen (river km 621.5). In spring and summer small amounts were detected even at Reiherstieg-Vorhafen (river km 615.5). These observations are well correlated with data of geochemical approaches, indicating transport of high amounts of suspended solids of marine origin during low water discharge periods up to river km 623^25,26^. The elevated number of sequences associated with the *Nitrosomonas marina*-lineage in spring 2014 might be explained by an unusual low water discharge and a resulting increase of tide-dependent influences (the so called “tidal pumping”) within the respective season (Figures S3 and S4).

The four species which represent the *Nitrosomonas europaea*/*Nitrosomonas mobilis*-lineage, *N. eutropha* and *N. halophila* and the two eponymous ones are all characterized as halotolerant or moderately halophile. They also reveal a considerable tolerance against increasing ammonia concentrations and low substrate affinities (high Ks values of the ammonia oxidizing system). According to these features the majority of isolates were obtained from more or less eutrophicated habitats such as sewage disposal plants. Only few strains originated from freshwater or estuarine habitats. Cultivation independent approaches demonstrated species of this lineage to be most dominant in WWTPs, as well^76,77^.

Sequences affiliated with the *Nitrosomonas europaea/Nitrosomonas mobilis*-lineage, have been identified to occur predominately at Sandauhafen. Approximately 1 km downstream this site, Germany’s largest wastewater treatment plant (WWTP) has its outlet into the Southern Elbe. In view of this, and the fact that the number of respective sequences decreases continuously with increasing distance from Sandauhafen, a link between the abundance of members of the *Nitrosomonas europaea/Nitrosomonas mobilis*-lineage and the outlet of the WWTP is very likely.

The *Nitrosomonas communis*-lineage is composed of two subunits. The first is represented by *N. communis* and three further species, not described by name. The second is formed by the species *N. nitrosa.* Though phylogenetically closely related both sublineages are characterized by clear differences regarding to their distribution patterns in nature. *N. communis* and its relatives prefer pH neutral, often agriculturally utilized soils. Accordingly, the four species of this sublineage have relatively low affinity for ammonia and lack ureolytic activity. *N. nitrosa,* by contrast, commonly is distributed in more or less eutrophicated freshwaters as well as in sewage of chemical-processing facilities^35,40^. While the affinity constants for ammonia are similar to those of the *N. communis* sublineage, *N. nitrosa* differs by its capability to use urea as ammonia source. In this study sequences affiliated with the *Nitrosomonas communis-*lineage were found to show by far the lowest relative abundances. Slightly raised values were recorded at Reiherstieg-Vorhafen. Considering ecophysiological properties and distribution patterns of this lineage we assume anthropogenic influences as a causal factor for this finding.

## Conclusion

There is strong evidence that the community composition of ß-AOB reflects environmental dynamics in the River Elbe Estuary. Shifts in AOB-lineage abundance face effects caused by the interaction of headwater discharge and tidal influences in the freshwater region of the estuary, especially pronounced as a result of extreme or exceptional events.

Against the background of our current knowledge on ecophysiological features and distribution patterns of ß-AOB and the findings of the present study we propose the following model conception: Members of the *Nitrosomonas oligotropha*-lineage are considered as typical inhabitants of the freshwater region of the Elbe Estuary while the mainly site specific occurrence of representatives of the *Nitrosomonas europaea/Nitrosomonas mobilis*- and the *Nitrosomonas communis*-lineage can be assigned to processes caused by anthropogenic activities in the Port of Hamburg area. Shifts in the relative abundance in favor of *Nitrosospira*, (with the exception of the marine environmental clones) indicate fluvial impact. Signatures affiliated with the *Nitrosomonas marina*-lineage, however, displays downstream influences. With this a promising microbial indicator system to get insights into the environmental dynamics within the River Elbe Estuary was proposed for the first time. Further studies are encouraged to examine whether this *amoA*-based approach can be also applied as a model for other ecosystems.

## Supplementary information

Supplementary Information 1.

Supplementary Information 2.

Supplementary Information 3.
