# Supplementary material for: Deep amoA amplicon sequencing reveals community partitioning within ammonia-oxidizing bacteria in the environmentally dynamic estuary of the River Elbe
Source: Sci Rep. 2020 Oct 13;10:17165. doi: 10.1038/s41598-020-74163-0 (PMC7555866; doi:10.1038/s41598-020-74163-0)
Supplement: Supplementary file 1 — Supplementary Information 1. [file 41598_2020_74163_MOESM1_ESM.pdf]

SUPPLEMENTARY MATERIAL

Deep amoA amplicon sequencing reveals community partitioning within ammonia-oxidizing bacteria in the environmentally dynamic estuary of the River Elbe

Malinowski, M.[1§]; Alawi, M.[2§]; Krohn, I.[1§]; Ruff, S.[1]; Indenbirken, D.[3]; Alawi, M.[3, 5]; Karrasch, M.[4]; Lüschow, R.[4]; Streit, W.R.[1]; Timmermann, G.[1] and Pommerening-Röser, A.[1]*

Figure S1: Canonical correlation analysis of the ammonia-oxidizing microbial composition and environmental parameters. Analyses are based on amoA gene amplicons.

Figure S2: Canonical correlation analysis of the ammonia-oxidizing microbial composition on OTU level and environmental parameters. Analyses are based on amoA gene amplicons.

Figure S3: Correlation between Cadmium content in suspended particulate matter (red) at Seemannshöft (km 629) (monthly samples, three value moving average) and head water discharge (blue) at Neu Darchau (km 536) (Source: FGG-Elbe, https://www.elbe-datenportal.de)

Tables

Table S1: OTU-table (see excel-file)

Table S2: environmental parameters (see excel-file)

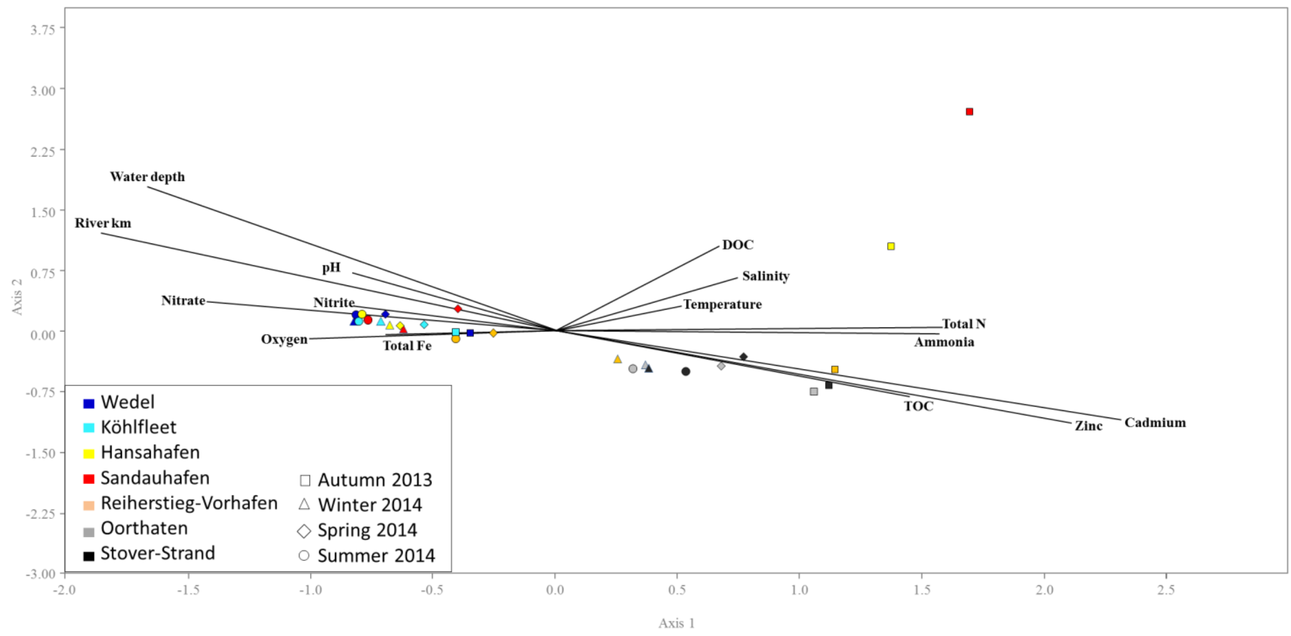

Figure S1: Canonical correlation analysis of the ammonia-oxidizing microbial composition and environmental parameters. Analyses are based on amoA gene amplicons.

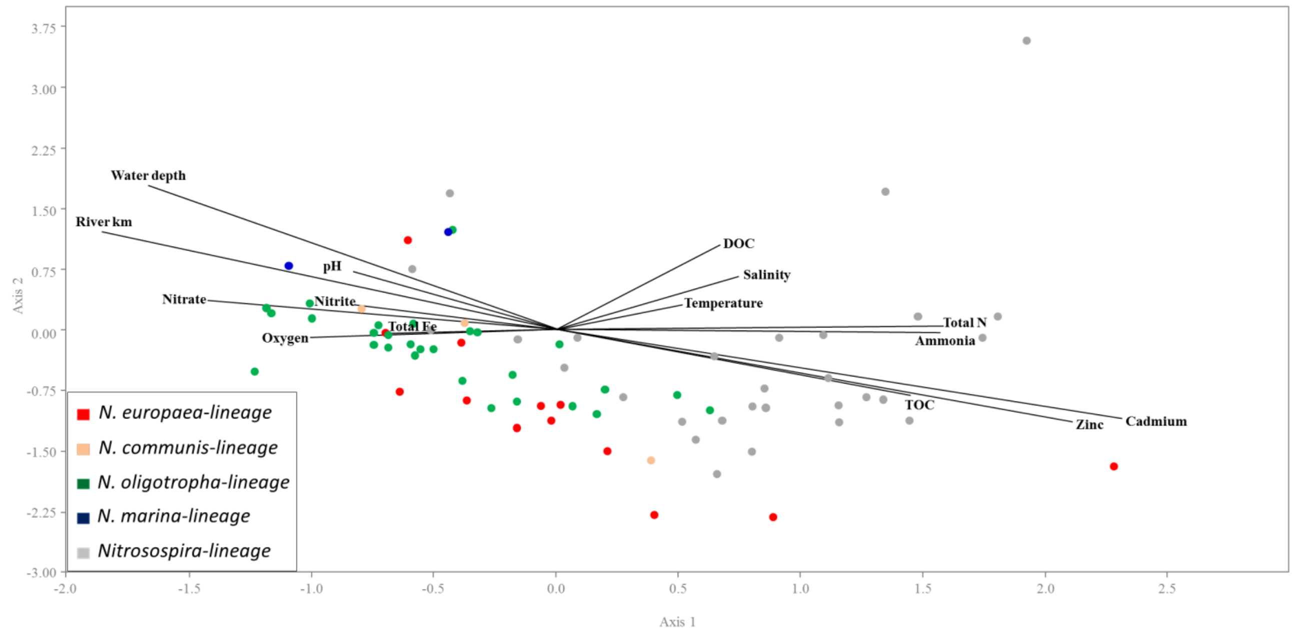

Figure S2: Canonical correlation analysis of the ammonia-oxidizing microbial composition on OTU level and environmental parameters. Analyses are based on amoA gene amplicons.

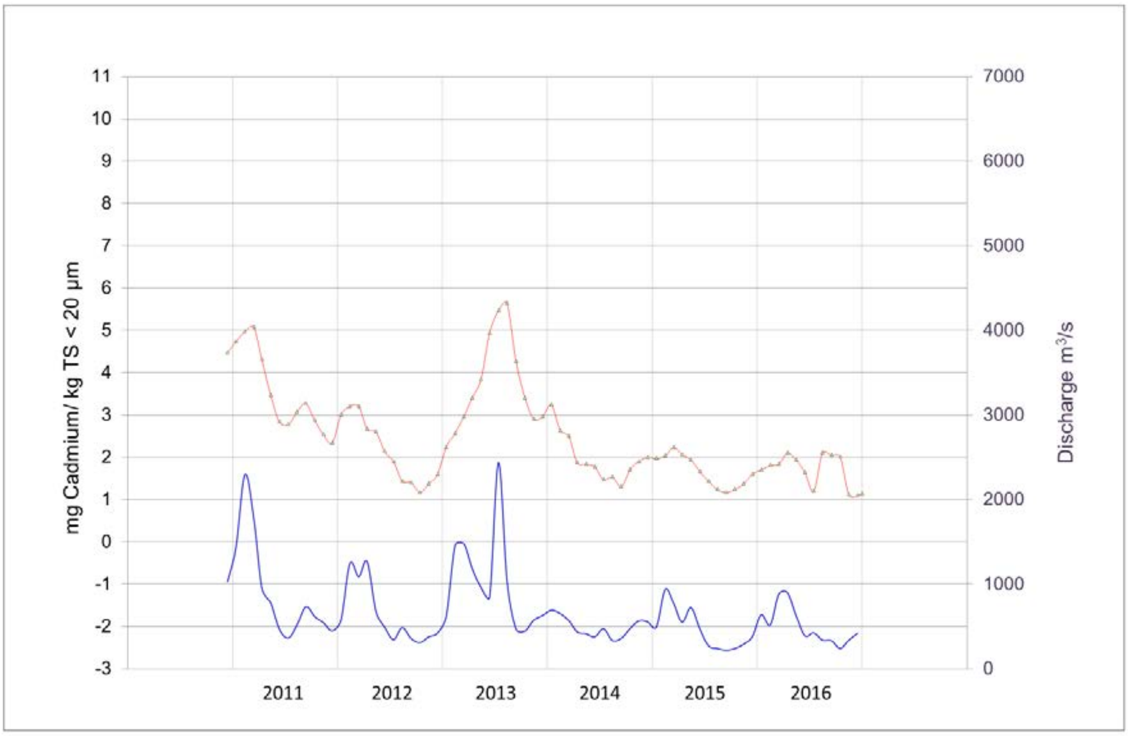

Figure S3: Correlation between Cadmium content in suspended particulate matter (red) at Seemannshöft (km 629) (monthly samples, three value moving average) and head water discharge (blue) at Neu Darchau (km 536) (Source: FGG-Elbe, https://www.elbe-datenportal.de)

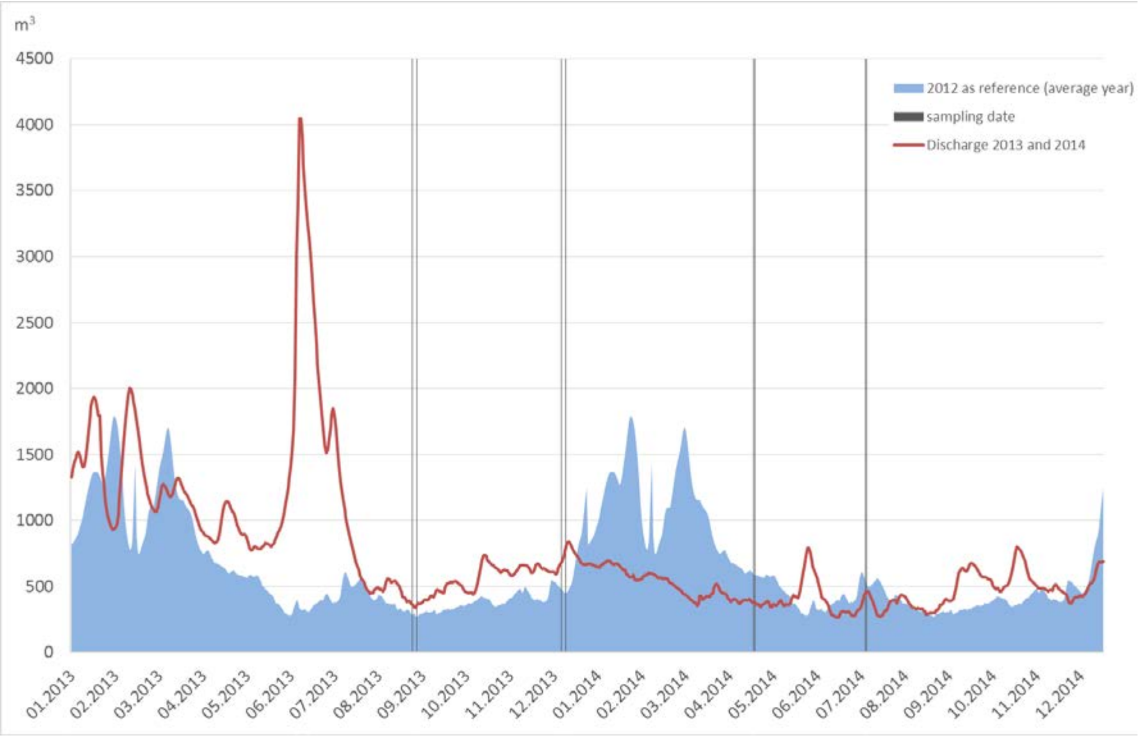

Figure S4: River Discharge at Neu Darchau (km 536) in 2013 and 2014 (red line). Vertical lines represent date of sampling. River Discharge in 2012 in blue as a reference (= average year). (Source: FGG-Elbe, https://www.elbe-datenportal.de
